# ^68^Ga-Trivehexin PET/CT uptake in malignant and fibrotic lung tissue: refining diagnostic applications

**DOI:** 10.1007/s00259-025-07193-3

**Published:** 2025-03-14

**Authors:** Serkan Kuyumcu, Dilara Denizmen Zorba, Zeynep Gözde Özkan

**Affiliations:** Department of Nuclear Medicine, Istanbul Faculty of Medicine, Istanbul University, Istanbul, Turkey

**Keywords:** ^68^Ga-Trivehexin, Lung cancer, Pulmonary fibrosis, Integrin, Αvβ6

We report a 55-year-old male with recurrent right lung adenocarcinoma who was initially diagnosed with left upper lobe adenocarcinoma (cT2acN0cM0, stage IB) four years ago. The patient opted for chemoradiotherapy at initial diagnosis, achieving significant tumour regression, with follow-up CT showing near-total reduction of the tumour in the left lung and mild post-radiation changes. Subsequently, the patient was lost to follow-up until when recurrence in the right lung was detected. Restaging with [^18^F]FDG PET/CT (Fig. 1 a, b,c) was performed as part of standard practice. Given its high specificity for epithelial malignancies, ^68^Ga-Trivehexin (Fig. 1 d, e,f) PET/CT, was performed (within one week) to assess thoracic and extrathoracic metastases. Both revealed uptake in the recurrent lesion (blue arrows). As bronchoalveolar lavage ruled out infection and malignancy, bronchiectasis and subpleural reticular density within the left lung as shown in CT images (Fig. 1 g, h) were attributed to post-radiotherapy fibrotic infiltrates (red arrows) which exhibited no [^18^F]FDG (Fig. 1 b, c) but ^68^Ga-Trivehexin uptake (Fig. 1 e, f).

**Fig. 1:**
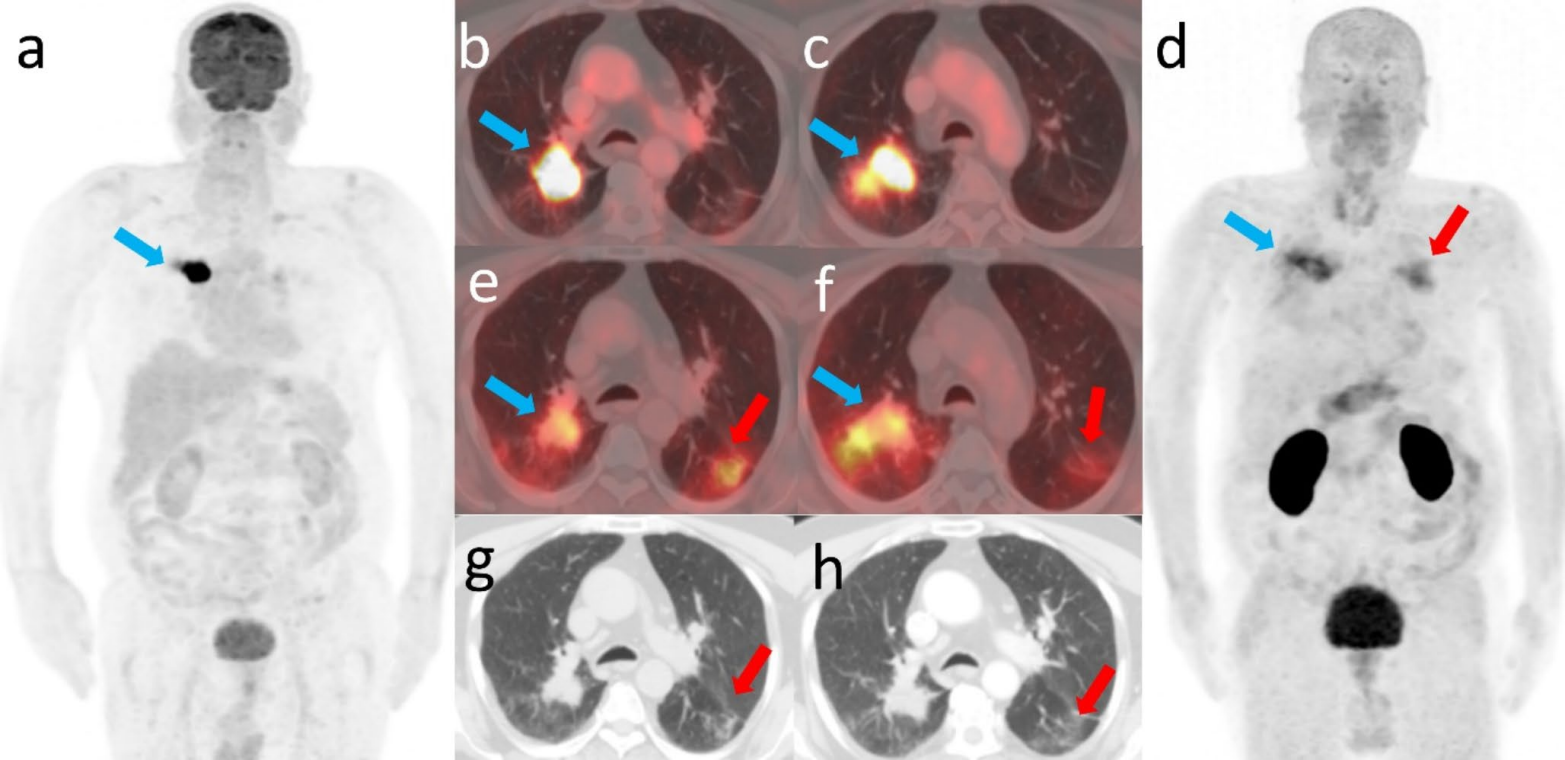
Figure 1

Unlike [^18^F]FDG PET/CT which may yield false positives in inflammatory conditions, the αvβ6 integrin receptor is more specific, potentially enhancing diagnostic accuracy in epithelial cancers [1, 2]. Nevertheless, the likelihood of non-malignant uptake, such as in parathyroid adenomas [3], warrants consideration. Similarly, this case demonstrates ^68^Ga-Trivehexin uptake in non-malignant fibrotic tissue, suggesting a potential for integrin-targeted imaging in assessing fibrotic diseases. Integrin αvβ6 significantly contributes to fibrogenesis [4], particularly in radiation-induced fibrosis and idiopathic pulmonary fibrosis, by activating latent transforming growth factor beta. Increased uptake of integrin-targeted PET tracers in fibrotic regions correlates with αvβ6 upregulation following radiation exposure [5]. The unexpected ^68^Ga-Trivehexin uptake presents interpretive challenges, but also offers a diagnostic opportunity, facilitating early detection and improving therapeutic strategies for conditions like radiation-induced pulmonary fibrosis or fibrotic lung diseases, where treatment options remain limited. These findings support that integrin imaging may provide insights into the dynamic processes of fibrosis, bridging diagnostic and therapeutic applications.

In conclusion, ^68^Ga-Trivehexin uptake in fibrosis offers a promising avenue for monitoring fibrotic diseases, warranting further research to optimize the clinical utility of integrin-targeted PET imaging.

## Data Availability

The datasets generated and/or analysed during the current study are available from the corresponding author upon reasonable request.
